# Supplementary material for: Research and design of an intelligent fish tank system
Source: PLoS One. 2023 May 4;18(5):e0285105. doi: 10.1371/journal.pone.0285105 (PMC10159123; doi:10.1371/journal.pone.0285105)
Supplement: Attachment — Submitted filename: Response to Reviewers.docx [file pone.0285105.s001.docx]

**Response to Reviewers**

Dear reviewers,

Re:Manuscript ID: PONE-D-22-12080 and Title: Design and implementation of intelligent fish tank system

Thank you for your letter and reviewers’comments our manuscript and allowing us to resubmit a revised copy of the manuscript.

Those comments are valuable and very helpful.we have read through comments carefully and have made corrections.Based on the instructions provide in your letter,we uploaded the file of the revised manuscript.Revision in the text are shown using yellow highlight for additions,and strikethrough font for deletions.The responses to the reviewer’s comments are marked in red . The major revisions and new analyses we have undertaken are summarized below and discussed in detail in the point-by-point responses.

**Specifically:**

**Q1:The contribution is not clear, and it does not discuss the methods and novelty.**

**Revision1:** The contribution we designed provides a convenient, time saving, scientific and efficient way for family fish farming, and combines theory with real life to solve the problems in life and make society develop. The description of "contribution" is modified and added in the abstract, introduction and conclusion of the article. The method is discussed in software design and experimental analysis. In the introduction, the novelty of this design is highlighted by comparing other designs.

**Q2:The authors should revise the manuscript to improve the quality of the writing flow and readability** of the text.

**Revision2:**The quality of the writing process and the readability of the text are improved by revising the overall manuscript.

**Q3:The contribution results are not appropriately explained.**

**Revision3:** The contribution results are explained in the conclusion.

**Comments to the Author:**

**Q1: Is the manuscript technically sound, and do the data support the conclusions?**

**Response:** The manuscript is technically reasonable, and the data supports the conclusion. The data and conclusion can be viewed through the experimental analysis and testing part of the article.

**Q2: Has the statistical analysis been performed appropriately and rigorously?**

**Response:** Statistical analysis method is not used.

**Q3: Have the authors made all data underlying the findings in their manuscript fully available?**

**Response:**Yes.

**Q4: Is the manuscript presented in an intelligible fashion and written in standard English?**

**Response:** Through the suggestions, we have improved the presentation and readability of the article, which is written in standard English.

**Review Comments to the Author:**

**Reviewer #1: Paper summary**

**The authors propose an IoT system to monitor the water temperature, pH and light fish tanks. Additionally, the system automatically adjusts the temperature, pH and feeding for the fish tank.
Overall, I believe that the system evaluation methods have not been clearly reported, the results are not appropriately explained, and interpretation of the findings is missing.**

**Response:**We are grateful for the suggestion. The system evaluation method adds specific methods to the summary, and the missing part of the result interpretation is described again in the conclusion.

**Major issues:**

**1.Abstract
a. The abstract is not clear, and it does not summarise the manuscript appropriately. Especially regarding the methods.**

**Response:**Thanks for your suggestion. The manuscript has been revised in the summary section, and the detailed methods used are listed.

**2.The system design is not entirely clear. Specifically, I have the following major issues:**

**a. The overall structure of intelligent fish tank remote monitoring system: Overall, the section lacks clarity and does not give an overview of the system. It would be helpful to give an overview of the design components: main controller, sensors, actuator/executing agency.**

**Response:** We are grateful for the suggestion. We have added the specific models and functions of the main controller, sensor, actuator/actuator to the overall structure of the intelligent fish tank remote monitoring system.

**b. Figure 8: The text is hard to read. From this diagram it seems like the pH is checked only if the light is suitable, but not if the light is unsuitable. Is this the case? And if so, why? Also, the temperature checking is missing. Also, why does this system have a start and an end? Does the user have to request the monitoring to start, rather than it being an automated process? If so, it defeats the purpose of an IoT.**

**Response:**Yes, because the light intensity affects the PH value of the water, when the light is weaker, the less CO2 is dissolved, the PH value will rise. At this time, it is necessary to open the oxygen enrichment equipment to increase the amount of CO2 dissolved to reduce the PH value until the PH value reaches the optimal value.The light intensity judgment in Figure 8 is a temperature check. The light intensity directly affects the temperature, so it is unnecessary to set a temperature check.I think there is no problem with the beginning and end of this system. This process is also an automated process and does not violate the purpose of the Internet of Things.

**c.The filtering algorithm section 4.4 is very unclear. It is unclear what approach is being taken and the reasons why, and what data was used.**

**Response:** Thanks for your suggestion. We combine the first-order lag filtering method with the median value average filtering method. Both methods have a good inhibition effect on periodic interference and are suitable for occasions with high fluctuation frequency. However, the first-order lag filtering method will be subject to occasional pulse interference, causing sampling value deviation. The median value average filtering method can solve this problem. In this process, we used the temperature, light intensity and water PH value data collected by the sensor.

**d.Figure 1:The authors should redraw the figure with English text.**

**Response:** We redraw Figure 1 in the manuscript in English.

**3. A methods section in which the system evaluation is described is missing, and thus it is not clear how the system is tested and evaluated.**

**Response:** We re described the method of system evaluation in the experimental analysis and testing part of the manuscript.

**4.The relevance of the Data Complexity Analysis Section 4.5 is unclear, as results for system speed are not provided.**

**Response:** We deleted this part.

**5. In the results, the figures and results lack explanation and interpretation. Additionally, they are missing axis units.**

**Response:** We have added relevant explanations and descriptions and relevant axis units.

**6. A discussion section to interpret the results and compare them to the current literature is missing. In this section, the limitations of the current work should be explored, and recommendations for future research should be made.**

**Response:** The differences between the results of this design and other designs have been compared in the introduction, so it is not necessary to compare again. The limitations of the current work and suggestions for future research have been added in the conclusion.

**7. I also have concerns regarding the accuracy and relevance of the refences provided. Major issues are: a.L69: Reference [13] refers to a study on Covid-19. Is that the correct reference?**

**Response:** We deleted this literature .

**b.L98-99: Reference [15] talks about an IoT for bedding monitoring using a pressure and piezoelectric sensor. So it is not clear how water temperature, pH and light can be measured from that.**

**Response:** Thank you for carefully reading this literature and making suggestions. We are similar to the author in that we use sensors to detect the required data and use the Internet of Things technology. The difference is that we use other types of sensors.

**8. The authors should revise the language to improve readability. I advise the authors work with a writing coach or copyeditor to improve the flow and readability of the text.**

**Response:** We revised the article to improve the smoothness and readability of the text.

**Additional issues:**

**1.The in-line reference placement is unclear. My suggestion is to move numbers before punctuation and right next to author name when used in-text.**

**Response:** Thank you for your suggestion. We have modified it.

**2.The authors should be consistent with how they write pH (many times spelled as PH).**

**Response:** Thank you for your suggestion. We have unified the way of writing.

**3. Introduction**

**a.L22-27: It is good to state the reasons why one would want to design an automated fish tank, but this paragraph does not do this in a clear manner.**

**Response:** We revised and explained the reason in the introduction of the manuscript.

**b.L35: Reference missing for Guo et al.**

**Response:** We supplemented the missing literature.

**c.L41-42: The authors say that the app display is simple, but in my view, there is nothing wrong with that. Further explanation would be useful.**

**Response:** The APP display interface is simple, and there is no real-time environmental parameter monitoring display.

**d.L47-48: This sentence is unclear, and references are missing.**

**Response:** Relevant references are supplemented.

**e.L64: Mentioning fish farming is confusing in the context of at-home fish tanks.**

**Response:** Thank you for your correction. Under the background of family aquarium, we modified the part of fish farming to meet the requirements of users for intelligent management of aquarium.

**4.The overall structure of intelligent fish tank remote monitoring system**

**a.L68-60: It would be useful to provide a rationale into the choice of environment parameters. I.e., why is temperature, light and pH important for the fish?**

**Response:** Thank you for your suggestions. Fish is a temperature changing animal. The water temperature is the body temperature. The level of water temperature directly affects the metabolic activities of life. Appropriate light can promote the thyroid secretion of fish, which is beneficial to the growth and development of fish. The pH value suitable for aquaculture ranges from 7.5 to 8.4. If the pH value is lower than 7, the water body will be acidic, which will stimulate the gills of the fish, reduce the oxygen carrying capacity of the blood of the fish, affect the respiration of the fish, and thus lead to the decline of the physical fitness of the fish and reduce the resistance.

**b.L74: A LED screen is mentioned only once in the paper, and is not visible on the tank in figure 12.**

**Response:** We have deleted the content of the LED screen, which is in the lower left corner of Figure 12

**5.System hardware design**

**a.L81: The authors should use consistent numbering for headings.**

**Response:** Thank you for your correction.We revised the manuscript and used consistent title numbers.

**b.L86: The authors say the STM32 is relatively cheap. Can the authors provide details?**

**Response:** At present, this conclusion is wrong. The price of chips on the market is rising, which is also the part we seek to improve, so we have deleted this part.

**c.Figure 2: Is the board custom-made or provided with the microcontroller? It would also be helpful to label each component for clarity.**

**Response:** Thank you for your suggestions. The circuit board is designed by ourselves. We have re marked the components of the circuit board on the manuscript.

**d.Figures 3,4,5,6,7: It would be helpful to also provide a high-level diagram of how the sensors are integrated within the system.**

**Response:** Thank you for your suggestions. We use the circuit diagram to show the function of the sensor in the manuscript.

**e.L125: Please define the acronym AT.**

**Response:** Thank you for your suggestions. AT means attention, we have already indicated it in the manuscript.

**f.L126: Please define the acronym MQTT.**

**Response:**MQTT means Message Queuing Telemetry Transport, we have already indicated it in the manuscript.

**g.L130: Unclear what reference [17] is used for.**

**Response:** The principle of the design circuit in this document is the same as that of our drive circuit, which is revised as the 15th document.

**6.The software design of intelligent fish cylinder system**

**a.L154: Unclear what reference [19] is used for.**

**Response:** We requoted a document in the manuscript

**b.L163: Unclear what reference [24] is used for.**

**Response:** We deleted this document.

**c.Figure 9: The text is hard to read.**

**Response:** This is the program flow chart. I don't know which points I can't understand.

**d.L171: Unclear what reference [21] is used for.**

**Response:** We deleted this document.

**e.Figure 10 is exactly the same as figure 8.**

**Response:** We replaced the correct picture in the manuscript.

**f.L181: Unclear what reference [26] is used for.**

**Response:** We deleted this document.

**g.L184-199: Please define the parameters Y1, Y2, X, alpha. Are a and alpha the same parameter?**

**Response:** Thank you for your suggestions. We redefined relevant parameters in the manuscript. Yes, they are the same parameter.

**h.L207: Why is the complexity O(N2) if there are no quadratic terms?**

**Response:** We deleted this part of the manuscript.

**7.Experimental analysis and testing**

**a.Figure 14: Units on the axis are missing.Also unclear what data is being measured and reported. Also, how is stability defined?**

**Response:** Thank you for your correction. We re marked the units represented by the number axis on the diagram. Test and report the data of temperature, PH value and light intensity detected by the sensor. Stability is the result of comparison test between actual sampling data and reference data.

**b.Figure 15:As the paper is in English, the figures also should be in English.**

**Response:** We corrected this in the manuscript.

**c.Figure 16, 20 and 22: Units on the axis are missing. Also unclear what is being measured and reported.**

**Response:** We added the units missing from the number axis in the manuscript.

**d.Figure 17, 19 and 21: These figures are not of high resolution and therefore hard to read.**

**Response:** We replaced clearer pictures in the manuscript.

**e.Figure 18:More explanation is needed. What is "real time" and what is "standard"? Units are missing.**

**Response:** Thank you for your suggestions. Real time means the data collected during operation, and standard means reference data. We have supplemented the digital axis unit of the picture in the manuscript.

**Reviewer#2:This paper lacks a scientific contribution. The methodology and results discussion are flawed.**

**Figure 1 should be translated into English. Otherwise, the reader cannot read the figure since this is a worldwide reader (international target of the journal). How to validate the economic benefit that increases by 3.5%. Lacking study and analysis of operation and comparison.The English must be written following the scientific journal writing.**

**Response:** Thank you for your correction.We modified this picture in the manuscript. Since the economic benefit of 3.5% increase cannot be verified, we deleted this point in the manuscript to ensure the accuracy of the research data. We reorganized the language logic to write this article.

Based on these comments and suggestions, we have made careful modifications to the original manuscript, and carefully proof-read the manuscript to minimize typographical and grammatical errors. We believe that the manuscript has been greatly improved and hope it has reached your magazine’s standard.

Sincerely.

Shaohua Fu
