# Supplementary material for: Research and design of an intelligent fish tank system
Source: PLoS One. 2023 May 4;18(5):e0285105. doi: 10.1371/journal.pone.0285105 (PMC10159123; doi:10.1371/journal.pone.0285105)
Supplement: Attachment — Submitted filename: Response to Reviewers.docx [file pone.0285105.s002.docx]

**Response to Reviewers**

Dear reviewers,

Re:Manuscript ID: PONE-D-22-31227 and Title: Design and implementation of intelligent fish tank system

Thank you for your letter and reviewers’comments our manuscript and allowing us to resubmit a revised copy of the manuscript.

Those comments are valuable and very helpful.we have read through comments carefully and have made corrections.Based on the instructions provide in your letter,we uploaded the file of the revised manuscript.Revision in the text are shown using yellow highlight for additions,and strikethrough font for deletions.The responses to the reviewer’s comments are marked in red . The major revisions and new analyses we have undertaken are summarized below and discussed in detail in the point-by-point responses.

**Specifically:**

**Q1: Reviewer #1: I appreciate the fact that from research a developed product comes out. However, the research component is one of the most important aspects of the product that is required in the research community; as a result, the development of the "fish tank system" that research proposed is missing a significant amount of the research components, which can be summarized as follows:
• While there is theoretical foundation of the algorithm of the data collected by the sensor, and a good description of an improved first-order lag average filtering algorithm, yet there is a need for the provision of the practical applications scenarios for the implementations, specifically in SECTION 4.
• While there is theoretical foundation for the system's usage of composite collection information, intelligent processing, chart data analysis, there practical implication from the finding of the study was not highlighted
• The end of the smart fish tank that deals with remote monitoring and control, as well as a visual data interface, lacks an in-depth analysis for the support of your development**

**Revision1:** First of all, thank you very much for your suggestions, and secondly, we have made the following changes based on your suggestions. The actual use scenario of this design is the indoor fish tank equipment, and the improved first-order lag average filtering algorithm is used in this equipment system. We use different algorithms to calculate the data samples collected in real time, and make their comparison tables and graphs in Section 4 of the manuscript，the comparison results show that the improved first-order lag average filtering algorithm has superiority. For the compound information collection method, we refer to the relevant literature in the introduction part, analyze its advantages and disadvantages, and improve the method that is beneficial to this system. For the intelligent processing method, we use improved algorithms and better-performing chips and sensors to process data in experiments. For the graph data analysis method, we draw graphs in Section 4 and 5 to compare data samples. By strengthening user feedback and surveys, we establish a close relationship with users of smart fish tanks and visual data interfaces, and collect user feedback and needs so that we can better understand their needs and develop new functions and improvements.

**Q2:** **Reviewer #2: The paper presents a real time monitoring fish tank system. In order to support the research on the designed system, it is recommended to the authors to include a summary table after the conducted research. While there is strong motivation, the performance issue highlighted is difficult to understand. Some paragraphs need to be improved with the correct conjunction. English improvement should be carried out before the final version submission.**

**Revision2:** Thank you for your suggestions on this article. According to your suggestions, we have made a hardware selection summary table at the end of section 3, and its parameters include the current and voltage of the hardware.

Based on these comments and suggestions, we have made careful modifications to the original manuscript, and carefully proof-read the manuscript to minimize typographical and grammatical errors. We believe that the manuscript has been greatly improved and hope it has reached your magazine’s standard. The author received no specific funding for this work. The authors declares that they have no conflict of interest.

Sincerely.

Shaohua Fu
